# Screening for peripheral neuropathy and peripheral arterial disease in persons with diabetes mellitus in a Nigerian University Teaching Hospital

**DOI:** 10.1186/s13104-015-1423-2

**Published:** 2015-10-04

**Authors:** Anthonia O. Ogbera, Olufunmilayo Adeleye, Babatunde Solagberu, Alfred Azenabor

**Affiliations:** Department of Medicine, Lagos State University College Of Medicine, Ikeja, Lagos, Nigeria; Department of Medicine, Lagos State University Teaching Hospital, Ikeja, Lagos, Nigeria; Department of Surgery, Lagos State University College Of Medicine, Ikeja, Lagos, Nigeria; Department of Medical Laboratory Sciences, University of Lagos, Idi-araba, Lagos, Nigeria

**Keywords:** Diabetes mellitus, Neuropathy, Peripheral arterial disease

## Abstract

**Background:**

Identifying the risk factors for diabetes mellitus related foot ulceration would save more limbs from amputation. This report focuses on the determining the burden of peripheral arterial disease and neuropathy in persons with diabetes mellitus (DM).

**Methods:**

This is a descriptive study carried out in the Diabetic Clinic of the Lagos State University Teaching Hospital in patients with DM who had no past/present history of foot ulceration. Biothesiometry was employed and ankle brachial pressure indices were measured to evaluate for neuropathy and peripheral arterial disease (PAD) respectively.

**Results:**

A total of 225 persons living with DM who met inclusion criteria were recruited consecutively over a 3 months period. Age range was 28–87 years with the mean [61.4 (10.8)] and median (63) years respectively. Patients symptomatic for neuropathy and PAD were 37 and 40 % respectively of the study population. An older age of >60 years and poor glycaemic control were potential predictors of neuropathy. Neuropathy and PAD occurred commonly in the seventh decade of life.

**Conclusion:**

Given the fairly high proportions of neuropathy and PAD in our patients with DM, we recommend that they be routinely examined in persons with DM.

## Background

Peripheral arterial disease, a pointer to the presence of atherosclerosis is oft reported in diabetes mellitus (DM). Potential sequelae of this finding in DM are diverse ranging from cardiovascular morbidity, the development of foot ulceration, increased risk for amputations and depending on severity, may result in death from any of the aforestated complications of DM. Neuropathy is a commonly documented complication of DM which may be asymptomatic but may lead to reduced quality of life in people who have painful neuropathy. Neuropathy is a well recognised risk factor of foot ulcers in persons with DM.

In order to reduce the burden of cardiovascular and foot complications of DM it is imperative to screen persons with DM for these entities. There are a variety of screening techniques which are currently in use and these include the evaluation of vibration perception threshold (VPT) using the biothesiometer and documentation of ABI to detect PAD [1]. In this Report, biothesiometry which has been shown to have good correlation scores with tuning fork, monofilament and ankle reflex was employed for the purposes of quantifying vibratory sense.

Diabetic peripheral neuropathy which is documented to be present in 60 % of diabetic persons confers a great risk of foot ulceration; microvascular disease and suboptimal glycemic control are also contributory. The prevalence of neuropathy was 76 % PAD was 6.3 % in patients without a prior history of foot ulceration as earlier reported in 2005 by Ogbera et al. [2]. The results of the Diabcare Report published in 2008, showed that peripheral neuropathy was documented in 59.2 % of persons with DM in Nigeria [3]. When compared to the 33 % by Kinnear in 1963 [4] and 48 % by Osuntokun et al. in 1971 [5], it is evident that peripheral neuropathy is on the increase in Nigeria.

People living with DM are at high risk for PAD which unfortunately is often associated with poor prognosis of foot ulceration. The determination of PAD using clinical symptoms is unreliable and the presence of neuropathy may be contributory to this scenario. Given the inconsistencies associated with the symptoms of PAD, the measurement of ankle-brachial pressure index (ABI) is an objective relatively simple, non-invasive and inexpensive technique employed in the evaluation of PAD. ABI test is widely used in the hospital setting not only to screen asymptomatic patients but also to diagnose patients with clinical symptom of PAD. In a Nigerian report on middle and elderly patients with DM, the prevalence rate of PAD was found to be 53 % [6]. Reports on PAD and neuropathy from Nigeria have hardly assessed these entities objectively. Currently, health care providers in Nigeria are better equipped to detect neuropathy and PAD in a more objective manner than in the past two decades.

This study was done to document the burden of PAD and neuropathy in persons with DM.

## Methods

This is a descriptive Study carried out at the Diabetes Clinic of the Lagos State University Teaching Hospital (LASUTH), Ikeja, Nigeria. This is a 750 bed hospital upgraded in 2001 from a General Hospital to a teaching hospital. It is located in the capital of Lagos State with highly subsidized health care costs as a result of governmental policy.

Consenting patients were recruited consecutively over a 3-month period. All persons living with DM aged more than 20 years and who had no past histories of foot ulceration, were recruited into the study. Ethical approval was obtained from the Lagos State University Teaching Hospital.

*Demography* validated interviewer administered questionnaires were used to obtain histories pertaining to clinical characteristics of DM status and complications of neuropathy and PAD, smoking, alcohol and co-morbidities such as hypertension. Long duration of DM means >10 years and older age referred to >60 years.

The anthropometric indices measured included waist circumference, height and body weight, and the body mass index (BMI) in weight/height^2^ (kg/m^2^). Waist circumference was determined by applying a tape measure to the midpoint between the inferior margin of the last rib and the crest of the ilium. The blood pressure was measured both in supine and upright positions.

A biothesiometer was used to determine semi quantitatively vibration perception thresholds in a standardized manner by a single observer. The biothesiometer probe, which vibrates at an amplitude proportional to the square of the applied voltage, was applied perpendicularly to the test site with a constant and firm pressure. Subjects were initially familiarized with the sensation by holding the probe against the distal palmar surface of hand. VPT was then measured at the distal plantar surface of great toe of both the legs. The voltage was slowly increased at the rate of 1 mV/sec and the VPT value was defined as the voltage level when the subject indicated that he or she first felt the vibration sense.

DM Neuropathy referred to a vibration perception threshold values of more than 15 mV with or without the presence of neuropathic symptoms [7].

A hand held Doppler equipment with a frequency of 12 MHz was used to document the ABI. The ABI was performed by measuring the systolic blood pressure from both brachial arteries and from both the dorsalis pedis and posterior tibial arteries after the patient has been at rest in the supine position for 10 min. We used a standard blood pressure cuff at the ankle. We began recording the pressures with the right arm, then the right leg, then the left leg, and finally the left arm.

The higher systolic blood pressure value was used as the numerator of the ABI in each limb. (An ABI of >1.30 denoted incompressibility of the vessels and persons with such readings were excluded from the analyses). The diagnostic criteria for PAD based on the ABI were interpreted as follows:

Normal if 0.91–1.30, Mild obstruction if 0.70–0.90, Moderate obstruction if 0.40–0.69, Severe obstruction if <0.40, Poorly compressible if >1.30 [8].

HbA1c was assayed using a fully automated Boronate Affinity assay for the determination of the percentage of hemoglobin A1C (HbA1c %) in whole blood. Poor glycaemic control consisted of glycosylated hemoglobin (Hba1c) levels of ≥7 % [9].

Hypertension was said to be present in persons known to have a history of elevated blood.

Fasting blood samples were taken for the determination of four parameters of the lipid profile viz total cholesterol (TCHOL), high density lipoprotein cholesterol (HDL-C), and triglyceride (TG). Total cholesterol assay was done using a modified method of Liebermann-Burchard, HDL-cholesterol by precipitation method and TG was estimated using a kit employing enzymatic hydrolysis of TG with lipases [10]. LDL-C was calculated using the Friedwald’s formula [10] LDL = (TCHOL − HDL-C) − TG/5 when the values of TG were less than 400 mg %. Abnormal lipid profile consisted of the following abnormalities either singly or in combination; triglyceride (TG) levels ≥150 mg %, high density lipoprotein cholesterol (HDL-C) (for men ≤40 mg % and women ≤50 mg %), low density lipoprotein cholesterol (LDL-C) ≥100 mg % [10].

Statistical analyses were performed with SPSS version 15. A multiple regression analysis with PAD as the dependent variable in conjunction with multiple independent variables was used to evaluate for possible predictors of PAD. Independent sample Student’s t test was used to compare quantitative data. χ^2^ was used to test for differences in proportions. A p value of ≤0.05 was deemed significant.

## Results and discussion

### Results

A total of 225 persons living with DM met the inclusion criteria. The age range was 28–87 years with the mean and median ages of the as 61.4 (10.8) and 63 years respectively. There were 163 (68 %) females giving a M: F ratio of 1:2.6. The mean age of the males was greater than that of females and the difference was statistically significant (63.6 vs 60.4, p = 0.03).

Other clinical parameters are shown in Table 1.

**Table 1:** Clinical characteristics of the DM patients evaluated for risk factors of foot ulceration

Parameter	Mean (SD)	Range
Age (years)	61.4 (10.8)	28–87
Duration of DM (years)	9.2 (7.6)	8–44
Body mass index (kg/m^2^)	27.6 (4.6)	17.5–42.5
Waist circumference (cm)	92.8 (12)	31–120
Haemoglobin (HbA1c) (%)	7.7 (2.1)	5–14

### Risk factors for DMFS

Neuropathy: Neuropathy was detected in 120 (56 %) of the study population. The proportions of females and males with neuropathy were comparable (M, 57 vs 56 % F, p = 0.9). Persons with neuropathy were older than those without neuropathy and this difference was statistically significant (63.9 (9) vs 58.2 (11) years, p = 0.0001). Positive symptoms of neuropathy ranging from burning nocturnal pain, pin and needle sensation were noted in 68 patients and negative symptoms of numbness was documented in 16 patients. Poor long term glycaemic control and being elderly as shown in Table 2 were some factors that were found to be predictive for the occurrence of peripheral neuropathy. More than half—47 (52 %) of the people with DM who had neuropathy also had PAD. A comparison of some clinical parameters between patients with neuropathy and those without neuropathy showed that these indices were comparable in both groups; BMI (27.5 vs 27.8, p = 0.6), waist circumference indices (93.1 vs 92.1, p = 0.6), glycosylated haemoglobin (7.7 vs 7.7 p = 0.9) and duration of DM (9.7 vs 8.6, p = 0.8).

**Table 2:** Predictors of peripheral neuropathy in patients with diabetes mellitus

Variables	OR	95 % confidence interval	p value
Age >60 years	2.6	1.4–4.8	0.02
Elevated HbA1c	2.0	1.1–3.80	0.002
Low HDL-C	2.1	0.4–8.3	0.3
Male sex	1.54	0.78–3	0.2
Elevated LDL-C	1.49	0.6–3.3	0.3
Duration of DM >10 years	1.29	0.7–2.6	0.4
Elevated TCHOL	1.1	0.4–2.5	0.8
Elevated TG	0.87	0.4–1.8	0.7
Overweight/obese	0.86	0.45–1.66	0.8
History of hypertension	0.72	0.36–0.72	0.3
PAD	0.73	0.42–1.3	0.3

### Peripheral arterial disease

The total number of persons with PAD was 90 (40 %). The proportions of females and males PAD were comparable (M, 43 vs 39 %, p = 0.9). The mean age of persons with PAD and those without PAD was comparable. The results of comparisons made for some clinical and biochemical parameters between persons with PAD and persons without PAD is shown in Table 3.

**Table 3:** Comparison of clinical parameters between subjects with PAD and those without PAD

Parameter	Patients with no PAD	Patients with PAD	p
Age (years)	61.2 (10.9)	61.5 (10.6)	0.8
Body mass Index (kg/m^2^)	27.8 (4.6)	27.5 (4.6)	0.8
HbA1c (%)	7.9 (2.2)	7.5 (1.8)	0.1
Duration of DM (years)	9.5 (7.7)	8.9 (7.6)	0.5
Waist circumference (cm)	93 (12.7)	92.5 (11.2)	0.3

**Fig. 1:**
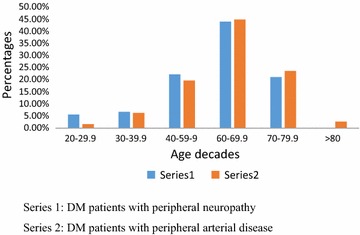
Distribution of neuropathy and peripheral arterial disease by age decades

DM associated neuropathy and peripheral arterial disease were prominent in the seventh decade of life. These results are shown in Fig. 1.

### Dyslipidaemia

**Fig. 2:**
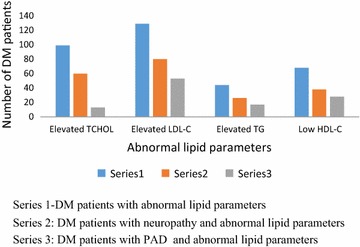
Distribution of abnormal lipid parameters in persons with DM

The proportions of the Study population with elevated total cholesterol, LDL-C, TG and low HDL levels were 99 (44 %), 44 (20 %), 129 (57 %) and 66 (29 %) respectively. A comparison of the distribution of abnormal lipid parameters between DM patients with PAD and DM patients with neuropathy is shown in Fig. 2.

## Discussion

There is a growing awareness amongst healthcare professionals on the importance of early detection of risk factors for diabetes mellitus foot ulceration especially because this complication of DM has been associated with a high disease morbidity and mortality in resource poor countries.

In this report, neuropathy was a commonly documented entity and painful neuropathy its prominent symptom was noted in about half of the patients studied. Impaired vibration sense is an early sign of neuropathy, hence its assessment is an important element of the neurological examination of the patient with diabetes [11]. We screened for neuropathy by means of biothesiometer, a recognised method of testing for vibration sensation. In a Study that compared the efficacy of the use of a biothesiometer with that of the tuning fork the biothesiometer was found not only to have comparable efficacy to the tuning fork but was more useful in large outpatient clinics [11]. In a Nigerian Study that reported the presence of neuropathy in persons with DM, the results showed that Bio-thesiometry detected neuropathy in 47.8 % of those with duration of DM <5 years and 100.0 % in those with duration of DM >15 years [12]. In our Report, neuropathy was diagnosed more in older persons with an odds ratio (OR) of 2.6 for above 60 years (nearly three higher risk). Although an association between ageing and the occurrence of neuropathy has been attested to in many pertinent studies [13–15] it is instructive to note that neuropathy may occur in any age group as we have shown in our findings. Other clinical parameters such as duration of DM, glycaemic control, lipid parameters did not differ between persons with DM neuropathy and those without neuropathy.

A possible relation between glycaemic control and DM complications has being pointed out by findings from the UK Prospective Diabetes Study (UKPDS) [16] and not surprisingly our results showed that poor glycaemic control, a readily modifiable risk factor is a possible predictor of DM neuropathy. The prevalence of neuropathy has been reported in a Canadian Study to increase with increasing glucose [17]. The potential role of hyperglycaemia in the pathogenesis of DM neuropathy has been explained by the polyol pathway theory and the glycosylation end product pathway [18]. This is because glucose uptake in peripheral nerves is not dependent on insulin and, high blood glucose levels in diabetes thus leads to high nerve glucose concentrations and this stimulates the polyol pathway [18]. Whilst some studies report duration of DM to be associated with DM related neuropathy, we found no such associations. This scenario may be explained partly by the fact that DMFS presents in 25 % as the initial manifestation of DM in our environment [19].

There is a dearth of Reports on detection on PAD using ABI from Africa. In a recent study from Uganda [20], the author noted that PAD was present in 39 % of persons with DM albeit with a male preponderance. These results are comparable to those of this Study. Our documented prevalence rate of PAD at 40 % is fairly high coming from Africa compared to earlier reported prevalence ranging from 1.7 to 28 % [21]. The results from the Report by Oyelade [6] demonstrates an upward rise of PAD in Nigerians as compared to data obtained some few decades back. Peripheral arterial disease is unfortunately often asymptomatic and remains underdiagnosed in the majority of persons with DM [22]. Given the limitations of relying on the clinical history or physical examination, an additional noninvasive test, ABI, is widely adopted for confirmation of a clinical diagnosis of peripheral arterial disease and its quantification. The ABI is a measure of the blood pressure in the arteries supplying legs relative to central, aortic pressure (approximated by measuring the blood pressure in the arm).

Barring the limitations of occasional high ABI indices that may be suggestive of incompressibility of the vessels, calculating the ABI for screening purposes is of benefit in detecting PAD even in the absence of foot ulcers. However it is imperative to note that the handheld Dopplers equipment does not give waveform pattern as and such, may fail to detect the PAD when Monckebergs sclerosis is present. We excluded persons with high ABI because of the potential of misleadingly high figures in detected PAD.. Hitherto, apart for research purposes, screening for PAD is not carried out routinely for persons living with DM in the hospital setting in hospitals in Lagos. Of interest also are our findings of comparable clinical and biochemical parameters between patients with PAD and patients without PAD. In this Report, PAD, was diagnosed albeit in a few persons even in persons who were aged less than 40 years. Hypertension and dyslipidaemia, though of fairly high prevalence rate, their role in the possible development of PAD and neuropathy could not be ascertained in this report.

### Limitations of the study

We did not document waveform patterns whilst examining for PAD.

## Conclusion

The burden of Peripheral neuropathy and peripheral arterial disease is high and may go undetected if not screened for. We recommend that PAD and neuropathy be screened for in diabetic clinics.

## Abbreviations

PAD: peripheral arterial disease
ABI: ankle brachial pressure index
HbA1c: glycosylated haemoglobin
TCHOL: total cholesterol
HDL-C: high density lipoprotein cholesterol
TG: triglyceride
LDL-C: low density lipoprotein cholesterol
BMI: body mass index
DMFS: diabetes mellitus foot syndrome
DM: diabetes mellitus
